# Multifocal FDG-avid pancreatic pseudomasses mimicking metastases during pembrolizumab for axillary recurrence of triple-negative breast cancer: a case report of immune-related pancreatitis

**DOI:** 10.3389/fonc.2026.1940160

**Published:** 2026-10-15

**Authors:** Paul Matte, Claire Charpignon, Vincent Servois, Virginie Huchet, Bruno Buecher, Caroline Malhaire, Jean-Yves Pierga, Luc Cabel, Delphine Loirat

**Affiliations:** 1 Department of Medical Oncology, Institute of Women's Cancers, Institut Curie, Paris, France; 2 Department of Gastroenterology, Institut Mutualiste Montsouris, Paris, France; 3 Department of Radiology, Institut Curie, Paris Sciences et Lettres (PSL) Research University, Paris, France; 4 Department of Nuclear Medicine, Institut Curie, Paris, France; 5 Departments of Genetics and of Surgical Oncology, Institut Curie, Paris Sciences et Lettres (PSL) Research University, Paris, France; 6 Department of Radiology, Laboratoire d'Imagerie Translationnelle en Oncologie (LITO), Inserm U1288, Institut Curie, Paris, France

**Keywords:** immune checkpoint inhibitors, immune-related adverse events, immune-related pancreatic injury, pancreatic insufficiency, pancreatitis, pseudomass, FDG PET/CT, triple-negative breast cancer

## Abstract

**Background:**

Immune checkpoint inhibitors (ICIs) are associated with a wide spectrum of immune-related adverse events (irAEs), reflecting enhanced immune activation. Pancreatic involvement is rare and typically presents as asymptomatic hyperlipasemia or diffuse inflammatory changes. Focal or multifocal mass-like presentations are uncommon and may mimic pancreatic malignancy, posing a significant diagnostic challenge.

**Case presentation:**

We report the case of a 48-year-old woman treated with Pembrolizumab and Paclitaxel for axillary recurrence of triple-negative breast cancer. After four cycles, FDG PET/CT demonstrated a complete metabolic response at the initial tumor site but revealed new multifocal hypermetabolic pancreatic lesions. Magnetic resonance imaging confirmed multiple pancreatic masses, raising suspicion of metastatic disease or dissociated response. Endoscopic ultrasound-guided fine-needle aspiration at two concordant pancreatic sites showed neutrophil-predominant inflammatory infiltration without malignant cells, consistent with immune-related pancreatic injury. The patient subsequently developed symptoms of exocrine pancreatic insufficiency, which improved after concomitant initiation of pancreatic enzyme replacement therapy and corticosteroid tapering, with the relative contribution of each not clearly distinguishable. Follow-up imaging demonstrated complete resolution of pancreatic lesions.

**Discussion:**

This case highlights a rare mass-forming (pseudotumoral) phenotype of ICI-related pancreatic injury (ICI-PI), presenting as multifocal FDG-avid pancreatic pseudomasses. Such findings may represent a major pitfall in PET/CT interpretation under immunotherapy, particularly in the context of otherwise favorable oncologic response. Histopathological confirmation is critical to distinguish immune-mediated inflammation from metastatic disease. This observation supports the concept of organ-specific immune dysregulation driven by ICIs and expands the radiological spectrum of pancreatic irAEs.

**Conclusion:**

Multifocal FDG-avid pancreatic lesions occurring during immunotherapy should raise suspicion for immune-related pancreatic injury (ICI-PI), especially in the absence of systemic progression. Early recognition is essential to avoid misdiagnosis and inappropriate treatment decisions.

## Introduction

Immune checkpoint inhibitors (ICIs) have significantly improved outcomes across multiple malignancies, including triple-negative breast cancer (TNBC). By enhancing T-cell–mediated antitumor immunity through programmed cell death protein 1 (PD-1), programmed death-ligand 1 (PD-L1), or cytotoxic T-lymphocyte-associated protein 4 (CTLA-4) blockade, these agents can also induce immune-related adverse events (irAEs) affecting virtually any organ system (1). Gastrointestinal irAEs are frequently observed with ICI, with diarrhea and colitis being the most commonly reported manifestations. These events occur in approximately 30–40% of patients treated with anti-CTLA-4 agents and in about 10–20% of those receiving anti-PD-1 therapies (2).

Pancreatic irAEs, collectively referred to as ICI-related pancreatic injury (ICI-PI), encompass a heterogeneous spectrum ranging from asymptomatic hyperlipasemia to clinically overt acute pancreatitis, imaging abnormalities, pancreatic atrophy, and exocrine insufficiency (3). Pancreatic irAEs are uncommon, with a reported incidence generally below 1% in meta-analyses (4–6). Most cases manifest as asymptomatic hyperlipasemia or acute interstitial pancreatitis without mass-forming lesions. Radiologically, a common pattern consists of diffuse pancreatic enlargement with homogeneous FDG uptake on PET/CT (7, 8). In contrast, focal or multifocal mass-like pancreatic lesions are rare and may closely mimic metastatic disease, creating a major diagnostic challenge, particularly in patients treated with ICIs in a curative or oligometastatic setting.

To our knowledge, only a limited number of biopsy-confirmed multifocal mass-like presentations have been reported. Here, we report a case of multifocal FDG-avid pancreatic pseudomasses occurring during Pembrolizumab (anti-PD-1) treatment for axillary recurrence of TNBC.

## Case presentation

A 48-year-old woman with no significant comorbidities, no relevant family history of breast or ovarian cancer or autoimmune disease, and a non-contributory psychosocial history was initially treated in 2020 for a stage I (T1cN0M0) triple-negative breast cancer (estrogen receptor-negative, progesterone receptor-negative, HER2-negative), with a Ki-67 proliferation index of 25%. Germline multigene panel testing did not identify a pathogenic variant in BRCA1 or BRCA2. After standard multimodal therapy (neoadjuvant dose-dense anthracycline-based chemotherapy, followed by breast-conserving surgery achieving a pathological complete response, adjuvant Paclitaxel, and radiotherapy), she remained disease-free until June 2024, when she developed an ipsilateral axillary lymph node recurrence. Biopsy confirmed TNBC with a PD-L1 combined positive score (CPS) of 15. She started weekly Paclitaxel combined with Pembrolizumab (200 mg intravenously every 3 weeks).

After four cycles (3 months), FDG PET/CT demonstrated a complete metabolic response of the axillary recurrence. However, new intense FDG uptake appeared in the pancreas, involving the head, body, and body-tail junction, with four distinct hypermetabolic foci. No other new hypermetabolic lesions were identified. Pancreatic metastases were considered, although the absence of additional systemic progression made this less typical (**Figure 1**). Magnetic resonance imaging (MRI) confirmed solid-appearing pancreatic lesions involving the head, body and body-tail junction. The largest lesion (38 mm) partially encased the superior mesenteric vessels (**Figure 2**). On MRI, several features favored an inflammatory rather than a neoplastic process: homogeneous T2 hyperintensity with only moderate diffusion restriction (rather than the marked restriction typical of adenocarcinoma or metastases), absence of significant arterial-phase enhancement with delayed but homogeneous portal-phase enhancement isointense to the adjacent pancreatic parenchyma, preserved patency of the encased superior mesenteric vessels without occlusion, and a normal-caliber Wirsung duct without significant upstream dilatation. The appearance of multifocal pancreatic lesions within three months under Pembrolizumab, despite complete metabolic response of previously involved tumor sites, primarily suggested autoimmune pancreatitis. However, the atypical multifocal pattern required considering a differential diagnosis of dissociated response with pancreatic metastatic progression.

**Figure 1:**
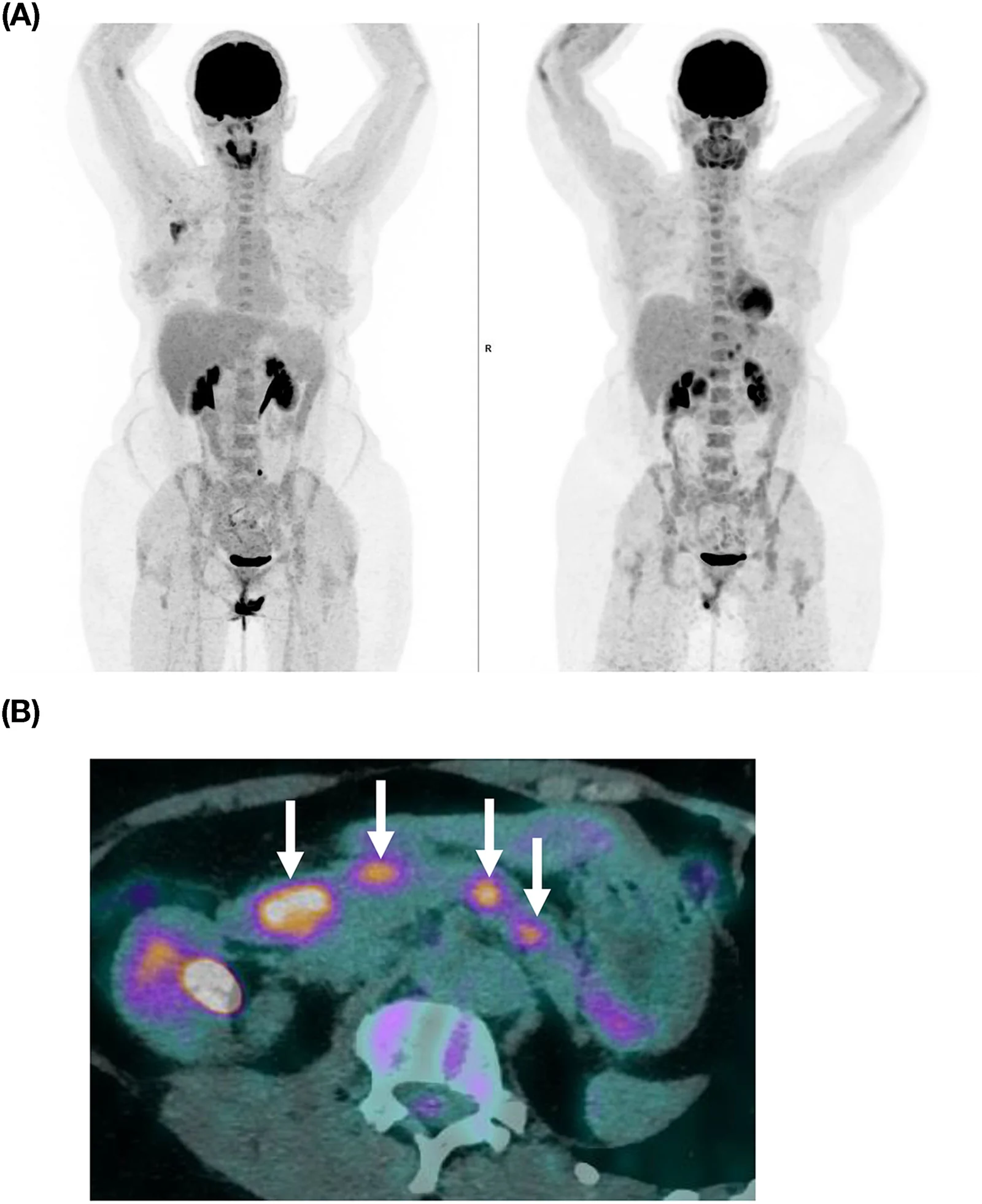
FDG PET/CT findings showing complete tumor response and new pancreatic uptake. **(A)** FDG PET/CT at recurrence (PET maximum intensity projection (MIP) view, left) shows several hypermetabolic right axillary lymph nodes. After 3 months on Pembrolizumab, FDG PET/CT (PET MIP view, right) shows complete metabolic response of right axillary involvement and appearance of pancreatic foci without any other suspicious uptake. **(B)** FDG PET/CT on Pembrolizumab (fused PET and CT, cross-section along pancreatic long axis) shows 4 hypermetabolic pancreatic foci (white arrows), respectively in the head, body, body-caudal junction and tail, with SUVmax ranging from 5.6 to 8.4.

**Figure 2:**
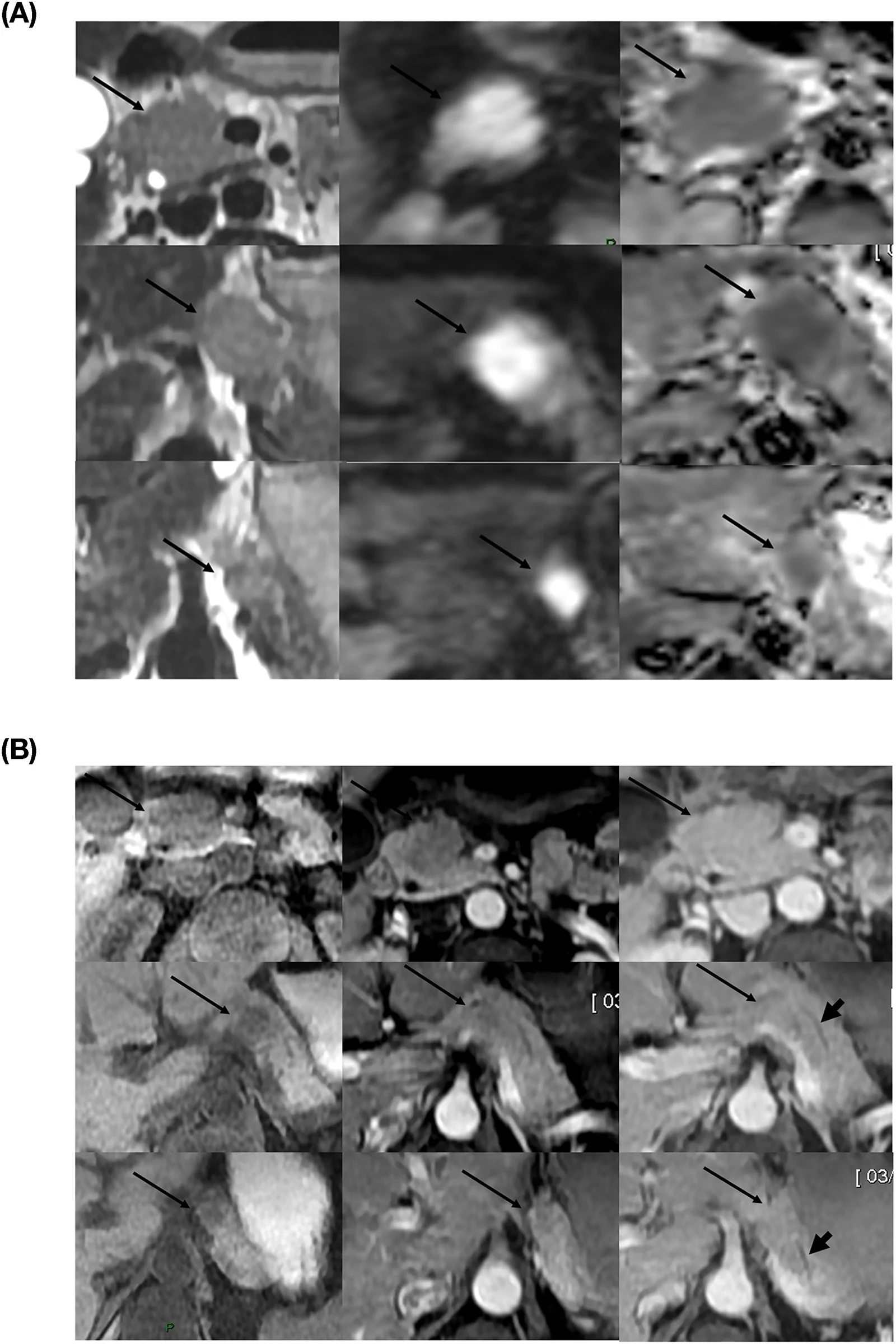
MRI features of multifocal pancreatic pseudomasses. **(A)** Axial MRI scans of the pancreas show three well-defined intrapancreatic masses (black arrows): the largest at the head of the pancreas (top line), one located in the body of the pancreas (middle line) and one located at the body-caudal junction of the pancreas (bottom line). All three lesions show a moderately hyperintense homogeneous signal on T2-weighted images (first column), hyperintensity on the diffusion-weighted sequence at b = 800 s/mm² (middle column) and moderate diffusion restriction on the ADC map (last column). **(B)** The dynamic T1-weighted series following injection of a gadolinium chelate shows the three intra-pancreatic masses (pancreatic head – top line, pancreatic body – middle line and pancreatic body-caudal junction – bottom line) (long black arrows). The three lesions appear hypointense compared with the normal pancreatic gland without contrast (first column), show no significant enhancement in the arterial phase 40 seconds after injection (middle column) and are homogeneously enhanced (isointense to the pancreatic gland) in the portal phase 70 seconds after injection (last column). Note the normal appearance of the Wirsung duct (short black arrow).

Biological workup showed minimal abnormalities, including borderline hypertriglyceridemia (1.59 g/L; normal <1.5 g/L) and grade 1 (CTCAE v5) lipase elevation (57 U/L; normal range 12–53 U/L). Blood glucose levels were normal. Endoscopic ultrasound revealed a globally hypoechoic and heterogeneous pancreatic gland with lobulated contours and partial narrowing of the main pancreatic duct. No clearly individualized tumoral nodules were visualized. EUS-guided fine-needle aspiration (EUS-FNA) was performed at two separate sites, the pancreatic isthmus and the pancreatic head, yielding tissue cores that were entirely included, after formalin fixation, in a cell-block and examined on hematoxylin-eosin-stained sections; no immunohistochemical panel was performed. Both samples showed superimposable histological features: pancreatic parenchyma with bands of fibrosis, interstitial edema, and a polymorphic inflammatory infiltrate predominantly composed of neutrophils and eosinophils with rare lymphocytes and plasma cells; focal acinar dystrophic changes with inflammatory exocytosis around pancreatic ducts, without granulomas; preserved islets of Langerhans; and no evidence of malignant proliferation at either site. This neutrophil-predominant pattern was considered, in the appropriate clinical context, morphologically consistent with an immune-mediated lesion related to immunotherapy, sometimes referred to as type 3 autoimmune pancreatitis, without histological evidence of malignancy. Pembrolizumab and Paclitaxel were discontinued.

After 6 weeks of Pembrolizumab discontinuation, the patient developed grade 2 diarrhea, characterized by pale, greasy stools and epigastric pain exacerbated by food intake, with a 5 kg weight loss and grade 2 hypokalemia. Abdominal CT showed no signs of colitis but revealed mild peripancreatic fat infiltration without changes in pancreatic lesions. Infectious workup, including stool cultures, Clostridioides difficile toxin assay, and viral studies, was negative. Autoimmune screening (anti-GAD65, anti-ZnT8, ANA, anti-dsDNA, ANCA, anti-CCP, and anti-myositis antibodies) was also unremarkable. Fecal elastase level was markedly reduced at 0.4 µg/g of stool, consistent with severe exocrine pancreatic insufficiency (defined as <100 µg/g).

Oral corticosteroid therapy (prednisolone 60 mg/day, approximately 1 mg/kg/day, in line with guideline-recommended dosing for grade ≥3 immune-related toxicity) was initiated to treat the presumed immune-mediated component of the presentation. Upper and lower endoscopy revealed no evidence of colitis. Duodenal, gastric, ileal, and colonic multiple random biopsies were normal, excluding immune-mediated colitis. Given the likely diagnosis of Pembrolizumab-induced exocrine pancreatic insufficiency, pancreatic enzyme replacement therapy (Pancreatin 50,000 IU three times daily) was initiated alongside corticosteroid tapering. Marked symptomatic improvement followed; because both treatments were started in close succession, their respective contributions to clinical recovery could not be firmly distinguished, although the rapid resolution of steatorrhea is most consistent with a direct effect of enzyme replacement.

One month later, the patient underwent right axillary lymph node dissection, which revealed three metastatic lymph nodes out of six, the largest measuring 11 mm in diameter. Given the recent pancreatic irAE, Pembrolizumab was not resumed; she instead received six months of adjuvant capecitabine and axillary radiation therapy as non-ICI-based adjuvant treatment. Follow-up FDG PET/CT performed three months later demonstrated complete resolution of pancreatic hypermetabolic lesions without new suspicious findings. No recurrence of gastrointestinal symptoms was observed after corticosteroid tapering.

## Discussion

The advent of ICIs has broadened the spectrum of irAEs, reflecting their widespread effects on immune activation. While pancreatitis is a recognized but rare irAE, its clinical presentation varies widely, ranging from asymptomatic enzyme elevation to severe acute inflammation (5, 9, 10). The reported incidence of ICI-PI is generally below 1%, with most cases presenting as subclinical lipase elevation or acute pancreatitis without radiographic change (5, 6). The occurrence of exocrine pancreatic insufficiency is rare, observed in 4% of ICI-PI cases (3, 11). In fact, the trials that led to the approval of Pembrolizumab in combination with chemotherapy for localized and metastatic TNBC did not report any cases of pancreatitis or elevated lipase levels (12, 13). Exocrine pancreatic insufficiency is increasingly recognized as a delayed manifestation of immune-related pancreatic injury and may occur even in the absence of marked enzyme elevation. Recognition is critical, as symptoms may mimic immune-related colitis but respond dramatically to pancreatic enzyme replacement therapy.

Terminology across this heterogeneous spectrum requires clarification. ICI-PI is an umbrella term encompassing asymptomatic pancreatic enzyme elevation, clinically overt acute pancreatitis, imaging abnormalities (including the mass-forming/pseudotumoral phenotype observed in the present case), pancreatic atrophy, and subsequent exocrine pancreatic insufficiency (3, 14). In our patient, serum lipase elevation was minimal (grade 1, 57 U/L) at the time the multifocal pancreatic lesions were identified, while clinically significant exocrine pancreatic insufficiency, weight loss, and hypokalemia subsequently developed, corresponding overall to a CTCAE grade 3 event driven predominantly by functional rather than biochemical severity. We therefore consider this case to represent ICI-PI with a mass-forming (pseudotumoral) phenotype, rather than ‘classical’ acute pancreatitis defined by marked hyperlipasemia. The histological finding of neutrophil-predominant inflammation without elevated serum IgG4 is suggestive of, but not synonymous with, the recently proposed ‘type 3 autoimmune pancreatitis’ phenotype of ICI-PI (15); given the limited amount of tissue obtained by fine-needle biopsy and the absence of immunohistochemical staining, we use this term descriptively rather than as a confirmed histopathological diagnosis. A recent large cohort study evaluating risk factors and long-term outcomes of ICI-PI similarly supports this broader classification and its heterogeneous natural history (14).

In our case, the emergence of new hypermetabolic lesions on PET-CT during ICI therapy, while previously involved tumor sites demonstrate metabolic response, raises two major differential diagnoses: dissociated response and irAE. Multiple pancreatic mass-like lesions are an unusual presentation of ICI-PI and have been reported in rare instances (8, 11).

On FDG PET/CT, ICI-PI most commonly presents as diffuse homogeneous hypermetabolism. However, focal or multifocal hypermetabolic patterns have been reported and may closely mimic metastatic disease (7, 16). Current EANM/SNMMI guidelines recommend systematic evaluation of organs prone to immune-related toxicity during PET interpretation under immunotherapy, including thyroid, pituitary, adrenal glands, and pancreas (17). In our case, isolated multifocal pancreatic uptake in the absence of other new hypermetabolic sites favored immune-mediated inflammation over systemic progression.

Pancreatic metastases are rare, accounting for approximately 2–3% of pancreatic malignant lesions in surgical and cytological series (18, 19). The diagnostic utility of endoscopic ultrasound-guided biopsy was critical in characterizing these lesions as inflammatory rather than neoplastic. However, the absence of malignant cells on FNA does not, by itself, fully exclude pancreatic metastasis; in this case, the diagnosis of immune-mediated pancreatic injury relied on the overall clinical course rather than on cytology/histology alone, the close temporal relationship with Pembrolizumab exposure, the complete metabolic response at the primary tumor site while new lesions appeared exclusively in the pancreas, the concordant inflammatory pattern at two independent biopsy sites, and the complete spontaneous resolution of all pancreatic lesions on follow-up imaging after ICI discontinuation, without any pancreas-directed oncologic treatment (3, 8).

The pathophysiology of ICI-PI remains incompletely understood. It is hypothesized that excessive T-cell activation triggered by ICIs leads to immune-mediated damage targeting pancreatic antigens, supported by immunohistochemical evidence of increased CD8+ T-cell infiltration in affected tissues (20, 21). The histological findings in this case (neutrophil-predominant inflammation without elevated IgG4) are consistent with, though not definitively diagnostic of, the recently described type 3 autoimmune pancreatitis phenotype of pancreatic irAEs (6, 15). Unlike classical IgG4-related autoimmune pancreatitis (type 1), type 3 autoimmune pancreatitis is not associated with elevated serum IgG4 levels and is characterized histologically by neutrophilic and CD8+ T-cell predominant inflammation. These findings suggest that ICI-PI may share pathophysiologic mechanisms with autoimmune pancreatitis, leading to chronic pancreatic alterations, including atrophy in some instances (3, 22).

Management in this case involved permanent discontinuation of Pembrolizumab (**Figure 3**). The decision to permanently discontinue Pembrolizumab, rather than to interrupt therapy with a view to rechallenge after recovery, was made by a multidisciplinary tumor board. It reflected both the overall severity of the presentation (weight loss, hypokalemia, and severe exocrine insufficiency, corresponding to a CTCAE grade 3 event) and the broader oncologic context: the patient was proceeding to axillary lymph node dissection followed by adjuvant capecitabine and radiotherapy, a treatment sequence expected to provide curative-intent locoregional and systemic control without requiring further immunotherapy. Rechallenge was therefore not considered, consistent with guideline recommendations to avoid ICI reintroduction after high-grade or organ-threatening irAEs when effective non-ICI treatment alternatives remain available (23, 24). Although corticosteroids are the mainstay of treatment for severe irAEs, their role in ICI-PI is debated, particularly for cases with mild biochemical abnormalities or asymptomatic enzyme elevation (3, 9). Given the patient’s mild biochemical alterations and lack of severe symptoms, a conservative approach was first adopted, consistent with recent recommendations favoring individualized management based on disease severity (23, 24). As detailed above, symptomatic improvement in our patient followed the concurrent initiation of pancreatic enzyme replacement therapy and corticosteroid tapering; the two interventions cannot be formally distinguished, although the pattern and rapidity of symptom resolution suggest that enzyme replacement played the dominant role in resolving steatorrhea and weight loss, while ICI discontinuation (with or without a contribution from corticosteroids) more plausibly accounts for the delayed radiological resolution of the pancreatic lesions themselves.

**Figure 3:**
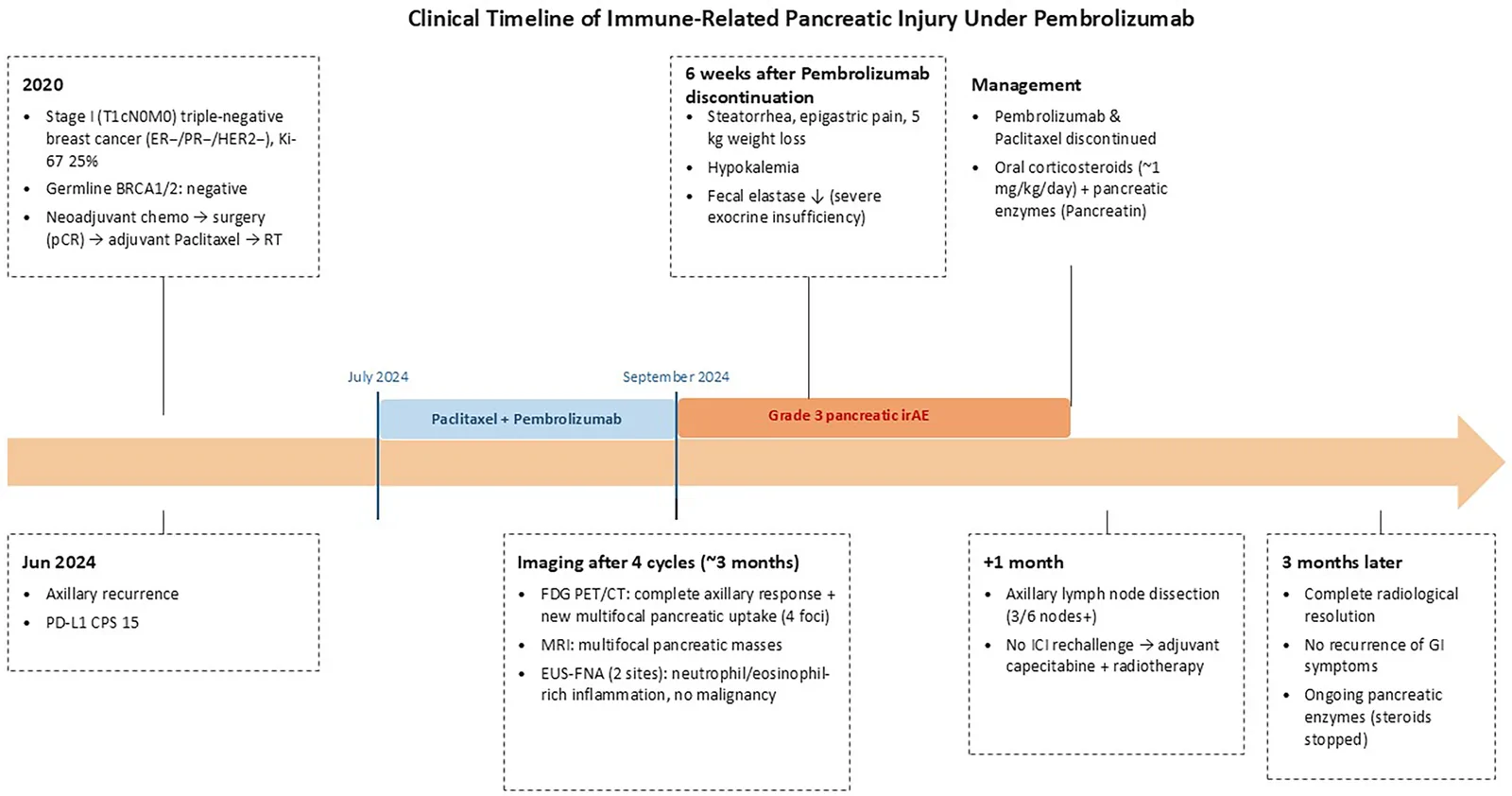
Clinical timeline of immune-related pancreatic injury under pembrolizumab. Timeline summarizing the patient’s oncologic history and the chronology of the pancreatic immune-related adverse event. 2020: initial diagnosis of stage I (T1cN0M0) triple-negative breast cancer, treated with neoadjuvant chemotherapy, surgery (pathological complete response), adjuvant chemotherapy, and radiotherapy. June 2024: ipsilateral axillary lymph node recurrence (PD-L1 CPS 15). July–September 2024: treatment with Paclitaxel plus Pembrolizumab. After 4 cycles (3 months): FDG PET/CT showed complete axillary metabolic response with new multifocal pancreatic FDG uptake; MRI confirmed multifocal pancreatic lesions; EUS-FNA showed inflammatory infiltration without malignancy. Six weeks after Pembrolizumab discontinuation: onset of grade 3 immune-related pancreatic injury with steatorrhea, epigastric pain, 5 kg weight loss, hypokalemia, and reduced fecal elastase, managed with Pembrolizumab discontinuation, oral corticosteroids, and pancreatic enzyme replacement therapy (Pancreatin). Three months later: complete radiological resolution, no recurrence, ongoing pancreatic enzyme supplementation without steroids.

Several case reports have described focal or mass-like presentations of immune checkpoint inhibitor–related pancreatitis, which may mimic pancreatic malignancy. A solitary pancreatic mass has been reported under Pembrolizumab (by abdominal ultrasound and MRI), leading to unnecessary surgical resection before a diagnosis of autoimmune pancreatitis was established (25). FDG PET/CT may also reveal focal hypermetabolic lesions, with complete resolution after corticosteroid therapy (7). In contrast, diffuse pancreatic uptake represents the more classical imaging pattern (26). Delayed presentations and atypical features such as capsule-like rim and subsequent pancreatic atrophy have also been described (22). Rare cases of multifocal pancreatic lesions mimicking malignancy have been reported, with diagnosis confirmed by endoscopic ultrasound-guided biopsy (27).

Our case is distinguished by multiple synchronous FDG-avid pancreatic pseudomasses occurring in the context of an otherwise complete metabolic response, highlighting a potential pitfall in PET interpretation under immunotherapy. The rarity of pancreatic masses in ICI-PI underscores the need for further research to better characterize this phenomenon. Additionally, the identification of predictive biomarkers remains an important research priority. For steroid-refractory presentations, agents such as rituximab, tacrolimus, or azathioprine have been reported as experimental, case-based therapeutic options, often extrapolated from other immune-mediated or autoimmune pancreatic conditions; evidence supporting their use specifically in ICI-PI remains limited to isolated case reports, and they should not be regarded as established treatment strategies (22, 28).

This report has several limitations. No immunohistochemical staining (e.g., CD8, IgG4) was performed on the pancreatic biopsy specimens, precluding definitive histopathological subclassification as type 3 autoimmune pancreatitis; the diagnosis therefore rests on a convergent clinical, radiological, and morphological picture rather than on a single confirmatory test. As a single case, the generalizability of this presentation is limited, and predictive factors for the mass-forming phenotype of ICI-PI remain unknown. The relative contributions of corticosteroids and pancreatic enzyme replacement therapy to symptomatic improvement could not be disentangled, as both were introduced in close succession. The main strengths of this report are the comprehensive multimodal work-up, sequential FDG PET/CT, MRI, and EUS-guided biopsy at two independent pancreatic sites with concordant findings, and the long-term follow-up confirming complete and durable radiological resolution, which together provide strong circumstantial evidence against metastatic disease and support an immune-mediated mechanism.

In conclusion, this case illustrates a rare but clinically significant manifestation of ICI-PI presenting as pseudo-tumoral pancreatic masses. It illustrates the need for multidisciplinary interpretation of new pancreatic findings arising during immunotherapy, integrating treatment chronology, response pattern, cross-sectional imaging, endoscopic assessment, pathology, and delayed functional outcomes.

## Data Availability

The original contributions presented in the study are included in the article/supplementary material. Further inquiries can be directed to the corresponding author.

## References

[1] Champiat S Lambotte O Barreau E Belkhir R Berdelou A Carbonnel F . Management of immune checkpoint blockade dysimmune toxicities: a collaborative position paper. Ann Oncol. (2016) 27:559–74. doi: 10.1093/annonc/mdv623 26715621

[2] Tandon P Bourassa-Blanchette S Bishay K Parlow S Laurie SA McCurdy JD . The risk of diarrhea and colitis in patients with advanced melanoma undergoing immune checkpoint inhibitor therapy: a systematic review and meta-analysis. J Immunother. (2018) 41:101–8. doi: 10.1097/CJI.0000000000000213 29401166

[3] Ashkar M Chandra S Vege SS Takahashi H Takahashi N McWilliams RR . Pancreatic involvement due to immune checkpoint inhibitors: a proposed classification. Cancer Immunol Immunother. (2023) 72:895–901. doi: 10.1007/s00262-022-03295-1 36161510 PMC10991382

[4] Su Q Zhang X-C Zhang C-G Hou Y-L Yao Y-X Cao B-W . Risk of immune-related pancreatitis in patients with solid tumors treated with immune checkpoint inhibitors: systematic assessment with meta-analysis. J Immunol Res. (2018) 2018:1027323. doi: 10.1155/2018/1027323 29971244 PMC6008648

[5] George J Bajaj D Sankaramangalam K Yoo JW Joshi NS Gettinger S . Incidence of pancreatitis with the use of immune checkpoint inhibitors (ICI) in advanced cancers: a systematic review and meta-analysis. Pancreatology. (2019) 19:587–94. doi: 10.1016/j.pan.2019.04.015 31076344

[6] Koseki M Nishimura Y Elias E Estaris J Chesta F Takaoka K . Pancreatitis in patients with cancer receiving immune checkpoint inhibitors: a systematic review and meta-analysis. Target Oncol. (2024) 19:867–77. doi: 10.1007/s11523-024-01098-1 39392549

[7] Das JP Halpenny D Do RK Ulaner GA . Focal immunotherapy-induced pancreatitis mimicking metastasis on FDG PET/CT. Clin Nucl Med. (2019) 44:836–7. doi: 10.1097/RLU.0000000000002692 31283599

[8] Hana C Rehman T Park K Uribe CC Aung PP Hunis B . Pancreatic adverse events in patients treated with immune checkpoint inhibitors. JGH Open: Open Access J Gastroenterol Hepatol. (2023) 7:204. doi: 10.1002/jgh3.12875 36968572 PMC10037034

[9] Townsend MJ Benque IJ Li M Grover S . Review article: contemporary management of gastrointestinal, pancreatic and hepatic toxicities of immune checkpoint inhibitors. Aliment Pharmacol Ther. (2024) 59:1350–65. doi: 10.1111/apt.17980 38590108

[10] Townsend MJ Liu M Giobbie-Hurder A Sack JS LeBoeuf NR Hodi FS . Pancreatitis and hyperlipasemia in the setting of immune checkpoint inhibitor therapy. J Natl Compr Canc Netw. (2023) 21:831–840.e3. doi: 10.6004/jnccn.2023.7034 37549912

[11] Jiang C Tang W Yang X Li H . Immune checkpoint inhibitor-related pancreatitis: what is known and what is not. Open Med. (2023) 18:20230713. doi: 10.1515/med-2023-0713 37273919 PMC10238808

[12] Cortes J Cescon DW Rugo HS Nowecki Z Im S-A Yusof MM . Pembrolizumab plus chemotherapy versus placebo plus chemotherapy for previously untreated locally recurrent inoperable or metastatic triple-negative breast cancer (KEYNOTE-355): a randomised, placebo-controlled, double-blind, phase 3 clinical trial. Lancet. (2020) 396:1817–28. doi: 10.1016/S0140-6736(20)32531-9 33278935

[13] Schmid P Cortes J Dent R McArthur H Pusztai L Kümmel S . Overall survival with pembrolizumab in early-stage triple-negative breast cancer. N Engl J Med. (2024) 391:1981–91. doi: 10.1056/NEJMoa2409932 39282906

[14] Akazawa Y Nosaka T Murata Y Tanaka T Takahashi K Naito T . Risk factors and long-term prognostic impact of immune related pancreatic injury in patients receiving immune checkpoint inhibitors. Front Immunol. (2025) 16:1590992. doi: 10.3389/fimmu.2025.1590992 40799644 PMC12339528

[15] Shirwaikar Thomas A Chari ST . Immune checkpoint inhibitor-induced (Type 3) autoimmune pancreatitis. Curr Gastroenterol Rep. (2023) 25:255–9. doi: 10.1007/s11894-023-00885-6 37845557

[16] Cho SY Huff DT Jeraj R Albertini MR . FDG PET/CT for assessment of immune therapy: opportunities and understanding pitfalls. Semin Nucl Med. (2020) 50:518–31. doi: 10.1053/j.semnuclmed.2020.06.001 33059821 PMC8201415

[17] Lopci E Hicks RJ Dimitrakopoulou-Strauss A Dercle L Iravani A Seban RD . Joint EANM/SNMMI/ANZSNM practice guidelines/procedure standards on recommended use of [^18^F]FDG PET/CT imaging during immunomodulatory treatments in patients with solid tumors version 1.0. Eur J Nucl Med Mol Imaging. (2022) 49:2323–41. doi: 10.1007/s00259-022-05780-2 35376991 PMC9165250

[18] Chen MY Zarrin-Khameh N Xu Y . Fine needle aspiration of pancreatic lesions focusing on secondary tumors with emphasis of metastatic breast cancer: a clinicopathological study with follow-up. Cancer Med. (2023) 12:5485–93. doi: 10.1002/cam4.5374 36281523 PMC10028048

[19] Adsay NV Andea A Basturk O Kilinc N Nassar H Cheng JD . Secondary tumors of the pancreas: an analysis of a surgical and autopsy database and review of the literature. Virchows Arch. (2004) 444:527–35. doi: 10.1007/s00428-004-0987-3 15057558

[20] Hori Y Naitoh I Naiki-Ito A Kawai T Yoshida M Kato A . Incidence of pancreatic injury and pancreatitis in patients treated with immune checkpoint inhibitors. Clin Transl Gastroenterol. (2024) 15:e00667. doi: 10.14309/ctg.0000000000000667 38088372 PMC11042772

[21] Liu Y Zhang H Zhou L Li W Yang L Li W . Immunotherapy-associated pancreatic adverse events: current understanding of their mechanism, diagnosis, and management. Front Oncol. (2021) 11:627612. doi: 10.3389/fonc.2021.627612 33732647 PMC7959713

[22] Kramer S van Hee K Blokzijl H van der Heide F Visschedijk MC . Immune checkpoint inhibitor-related pancreatitis: a case series, review of the literature and an expert opinion. J Immunother. (2023) 46:271–5. doi: 10.1097/cji.0000000000000472 37216403 PMC10405787

[23] Haanen J Obeid M Spain L Carbonnel F Wang Y Robert C . Management of toxicities from immunotherapy: ESMO clinical practice guideline for diagnosis, treatment and follow-up. Ann Oncol. (2022) 33:1217–38. doi: 10.1016/j.annonc.2022.10.001 36270461

[24] Fang W Gao Y Shi X Zhang X Zhou S Zhu H . Immune checkpoint inhibitors-related pancreatitis with fulminant type 1 diabetes mellitus: case report and literature review. Front Immunol. (2023) 14:1243773. doi: 10.3389/fimmu.2023.1243773 37841238 PMC10569069

[25] Inoue S Tsunoda Y Yamamoto K Okamoto H . Immunoglobulin G4-related autoimmune pancreatitis and hypopituitarism following immune checkpoint inhibitor therapy. Intern Med. (2025) 64:209–15. doi: 10.2169/internalmedicine.3591-24 38811225 PMC11802208

[26] Alabed YZ Aghayev A Sakellis C Van den Abbeele AD . Pancreatitis secondary to anti-programmed death receptor 1 immunotherapy diagnosed by FDG PET/CT. Clin Nucl Med. (2015) 40:e528–9. doi: 10.1097/RLU.0000000000000940 26284765

[27] Shi W Tan B Li Y Zhu L Feng Y Jiang Q . The diagnosis of immune-related pancreatitis disguised as multifocal lesions on MRI by endoscopic ultrasound-guided fine-needle biopsy: a case report. Front Immunol. (2022) 13:933595. doi: 10.3389/fimmu.2022.933595 36177047 PMC9513040

[28] Santoro A Masini S Cavina R Tronconi MC De Vincenzo F . Rituximab in steroid-refractory immune-related pancreatitis: a case report. Front Oncol. (2023) 13:1205720. doi: 10.3389/fonc.2023.1205720 37588091 PMC10425594

